# Maintain Your Brain: recent findings, future challenges

**DOI:** 10.1002/alz70860_103197

**Published:** 2025-12-23

**Authors:** Henry Brodaty, Tiffany Chau, Megan Heffernan, Jeewani Anupama Ginige, Michael Millard, Perminder S. Sachdev, Kaarin J. Anstey, Nicola T Lautenschlager, John J McNeil, Louisa Jorm, Anthony Maeder, Maria A. Fiatarone Singh, Michael Valenzuela

**Affiliations:** ^1^ Centre for Healthy Brain Ageing (CHeBA), UNSW Sydney, Sydney, NSW, Australia; ^2^ George Institute, Sydney, NSW, Australia; ^3^ Western Sydney University, Penrith, NSW, Australia; ^4^ St Vincent's Hospital, Sydney, NSW, Australia; ^5^ UNSW Ageing Futures Institute, Syndey, NSW, Australia; ^6^ The University of Melbourne, Parkville, VIC, Australia; ^7^ Monash University, Melbourne, VIC, Australia; ^8^ University of New South Wales, Sydney, NSW, Australia; ^9^ Flinders University, Adelaide, SA, Australia; ^10^ University of Sydney, Sydney, NSW, Australia

## Abstract

**Background:**

Despite multimodal interventions showing more promise in preventing cognitive decline in older persons, results have been mixed and most studies have been conducted in person. Online approaches are more scalable and feasible to deliver at a population level, but no multimodal online intervention has yet demonstrated efficacy. We aimed to reduce cognitive decline with ageing using an online package of interventions, *Maintain Your Brain (MYB)* delivered intensively for 12 months followed by monthly boosters for 24 months.

**Methods:**

Invitations were sent to people aged 55‐77 years from the 45 and Up study, a population‐based cohort study of one‐in‐ten people aged 45 years and older in NSW, Australia (*n* = 267,000). Participants were required to be eligible for >=2 of 4 modules addressing Physical Activity, Nutrition, Cognitive Activity and mental well‐being. Participants received modules based on their risks, with 1:1 randomized allocation to active personalised coaching modules (intervention) or static information‐based modules (control). The primary outcome was change in an online global cognitive score. Secondary outcomes included specific cognitive domains, module outcomes, dementia risk scores and cost.

**Results:**

Of 96,418 invitations issued, 14,064 (14%) consented, 11,026 (11%) were eligible and 6,104 (6%) completed all baseline assessments. Over three years, using ITT analysis, the intervention group improved significantly more in the global composite cognition z‐score ES=0.18 (*p* <0.001). Complex attention, executive function, learning and memory, physical activity, nutrition and depression (all *p* <0.001), and dementia risk (*p* = 0.007) all improved.^1^ Costs were balanced by health savings. Feedback from participants was positive.

**Conclusion:**

An online platform tailored to individuals’ risk factor profiles over three years significantly improved cognition decline in older adults. Despite MYB being effective, scalable, and potentially able to delay dementia onset and cost effective, attracting government or commercial interest to deliver an improved MYB version at a wider population level has proved challenging.

**Reference**

1. Brodaty H, Chau T, Heffernan M et al. An online multidomain lifestyle intervention to prevent cognitive decline in at‐risk older adults: a randomized controlled trial. Nature Medicine 2025 doi.org/10.1038/s41591‐024‐03351‐6

